# SARS-CoV-2 prolonged infection during advanced HIV disease evolves extensive immune escape

**DOI:** 10.1016/j.chom.2022.01.005

**Published:** 2022-02-09

**Authors:** Sandile Cele, Farina Karim, Gila Lustig, James Emmanuel San, Tandile Hermanus, Houriiyah Tegally, Jumari Snyman, Thandeka Moyo-Gwete, Eduan Wilkinson, Mallory Bernstein, Khadija Khan, Shi-Hsia Hwa, Sasha W. Tilles, Lavanya Singh, Jennifer Giandhari, Ntombifuthi Mthabela, Matilda Mazibuko, Yashica Ganga, Bernadett I. Gosnell, Salim S. Abdool Karim, Willem Hanekom, Wesley C. Van Voorhis, Thumbi Ndung’u, COMMIT-KZN Team, Richard J. Lessells, Penny L. Moore, Mahomed-Yunus S. Moosa, Tulio de Oliveira, Alex Sigal

**Affiliations:** 1 Africa Health Research Institute, Durban, South Africa; 2 School of Laboratory Medicine and Medical Sciences, University of KwaZulu-Natal, Durban, South Africa; 3 Centre for the AIDS Programme of Research in South Africa, Durban, South Africa; 4 KwaZulu-Natal Research Innovation and Sequencing Platform, Durban, South Africa; 5 National Institute for Communicable Diseases of the National Health Laboratory Service, Johannesburg, South Africa; 6 MRC Antibody Immunity Research Unit, School of Pathology, Faculty of Health Sciences, University of the Witwatersrand, Johannesburg, South Africa; 7 Centre for Epidemic Response and Innovation, School of Data Science and Computational Thinking, Stellenbosch University, Stellenbosch, South Africa; 8 HIV Pathogenesis Programme, University of KwaZulu-Natal, Durban, South Africa; 9 Division of Infection and Immunity, University College London, London, UK; 10 Center for Emerging and Re-emerging Infectious Diseases, University of Washington, Seattle, WA, USA; 11 Department of Infectious Diseases, Nelson R. Mandela School of Clinical Medicine, University of KwaZulu-Natal, Durban, South Africa; 12 Department of Epidemiology, Mailman School of Public Health, Columbia University, New York, NY, USA; 13 Institute of Infectious Disease and Molecular Medicine, University of Cape Town, Cape Town, South Africa; 14 Department of Global Health, University of Washington, Seattle, WA, USA; 15 Max Planck Institute for Infection Biology, Berlin, Germany

**Keywords:** SARS-CoV-2, HIV, advanced HIV disease, Beta variant, Delta variant, evolution, immune escape, neutralization, variants of concern

## Abstract

Characterizing SARS-CoV-2 evolution in specific geographies may help predict properties of the variants that come from these regions. We mapped neutralization of a SARS-CoV-2 strain that evolved over 6 months from ancestral virus in a person with advanced HIV disease in South Africa; this person was infected prior to emergence of the Beta and Delta variants. We longitudinally tracked the evolved virus and tested it against self-plasma and convalescent plasma from ancestral, Beta, and Delta infections. Early virus was similar to ancestral, but it evolved a multitude of mutations found in Omicron and other variants. It showed substantial but incomplete Pfizer BNT162b2 escape, weak neutralization by self-plasma, and despite pre-dating Delta, it also showed extensive escape of Delta infection-elicited neutralization. This example is consistent with the notion that SARS-CoV-2 evolving in individual immune-compromised hosts, including those with advanced HIV disease, may gain immune escape of vaccines and enhanced escape of Delta immunity, and this has implications for vaccine breakthrough and reinfections.

## Highlights

• An ancestral SARS-CoV-2 infection that persisted over 6 months evolved neutralization escape
• Evolved virus serological relationship is mapped to ancestral virus and key variants
• Evolved virus shows greatest escape from Delta-elicited immunity and least from Beta-elicited immunity
• Evolved virus substantially but incompletely escapes BNT162b2 vaccine-elicited antibodies

## Graphical abstract

**Figure:**
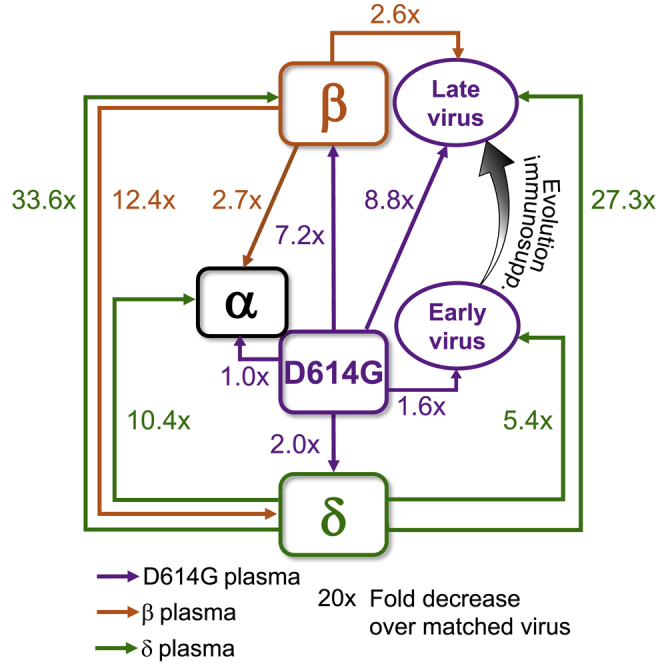

## Teaser

Cele et al. examine a SARS-CoV-2 infection persisting over 6 months, starting as ancestral virus but evolving various mutations found in Omicron and other variants. The evolved virus substantially but incompletely escaped BNT162b2-elicited immunity as well as neutralization by self-plasma and showed extensive escape from neutralization elicited by Delta infections.

## Main text

Neutralization is highly predictive of vaccine efficacy (Khoury et al., 2021, Earle et al., 2021). Some SARS-CoV-2 variants show decreased neutralization by vaccine-elicited immunity. This may make vaccines less effective at reducing the frequency of infection (Madhi et al., 2021). The Alpha variant shows relatively little escape (Garcia-Beltran et al., 2021a, Supasa et al., 2021, Planas et al., 2021a, Wang et al., 2021a). The Beta (Tegally et al., 2021, Cele et al., 2021a, Garcia-Beltran et al., 2021a, Wibmer et al., 2021, Zhou et al., 2021, Planas et al., 2021a, Wang et al., 2021a, Wang et al., 2021b), Gamma (Dejnirattisai et al., 2021), Lambda (Acevedo et al., 2021), and Mu (Uriu et al., 2021) variants show neutralization escape to different degrees from neutralization elicited by ancestral SARS-CoV-2 infection or vaccines. The Delta variant has evolved a transmission advantage over other SARS-CoV-2 strains (Mlcochova et al., 2021). It does not show a high degree of neutralization escape from plasma immunity elicited by ancestral strains, but it does show escape from Beta antibody immunity (Liu et al., 2021). As this work is being revised, the emergence of Omicron has led to a surge of SARS-CoV-2 infections worldwide. This variant has been shown by us (Cele et al., 2021b) and others (Andrews et al., 2021, Garcia-Beltran et al., 2021b, Cao et al., 2021, Lu et al., 2021, Rössler et al., 2021, Planas et al., 2021b) to have extensive escape from neutralizing immunity conferred by vaccines and by previous infections with other variants. It may also be much more transmissible, although whether this is inherent in a transmission advantage per contact or an increase in the number of contacts due to milder disease (Garret et al., 2021) is yet to be determined.

Escape from neutralization and enhanced transmission involves substitutions and deletions in the spike glycoprotein of the virus, which binds the ACE2 receptor on the cell surface (Barnes et al., 2020). Mutations associated with neutralization escape are found in the receptor-binding domain (RBD) (Barnes et al., 2020, Greaney et al., 2021a, Greaney et al., 2021b) and N-terminal domain of the spike (McCallum et al., 2021, Cerutti et al., 2021, Suryadevara et al., 2021, Chi et al., 2020). RBD mutations for the Beta variant include the K417N, E484K, and N501Y (see https://covdb.stanford.edu/page/mutation-viewer/). E484K and N501Y are shared with Gamma, which has the K417T instead of K417N. Alpha shares N501Y and in some cases E484K. Lambda has L452Q and F490S. Delta has L452R and T478K. Omicron has a multitude of mutations including G339D, S371L, S373P, S375F, K417N, N440K, G446S, S477N, T478K, E484A, Q493R, G496S, Q498R, N501Y, Y505H, and sometimes R346K. There are also extensive differences in the N-terminal domain (NTD). For example, Beta NTD substitutions include L18F, D80A, D215G, and a 241-243 deletion. In contrast, Delta has T19R, G142D, E156G, and Δ157-158. Omicron NTD mutations and deletions are A67V, Δ69-70, T95I, G142D, Δ143-145, N211I, and Δ212. In addition, Omicron has mutations in S2, including P681H—and D796Y, where the effect of the latter mutation is unclear.

An important consideration in some geographical areas is a high prevalence of co-infection of SARS-CoV-2 and HIV (Karim et al., 2021a). HIV can attenuate immunity to other infections. The mechanism may differ between co-infecting pathogens, but it is thought to involve a compromised antibody response because of depletion and dysregulation of CD4 helper T cells (Avelino-Silva et al., 2016, Ho et al., 1995). Antiretroviral therapy (ART) allows people living with HIV to avoid the worst consequences of HIV infection, which are the result of severe depletion of CD4 T cells (Murphy et al., 2001). However, lack of adherence to ART and development of drug resistance mutations leads to ongoing HIV replication, which, if left to persist for years, results in advanced HIV disease, which in turn leads to immune suppression (Murphy et al., 2001). We and others have recently shown that advanced HIV disease may lead to delayed clearance of SARS-CoV-2 and evolution of the SARS-CoV-2 virus (Hoffman et al., 2021, Karim et al., 2021b) similar to that with immune suppression due to other causes (Chen et al., 2021, Choi et al., 2020, Clark et al., 2021, Kemp et al., 2021, Weigang et al., 2021).

**Figure 1:**
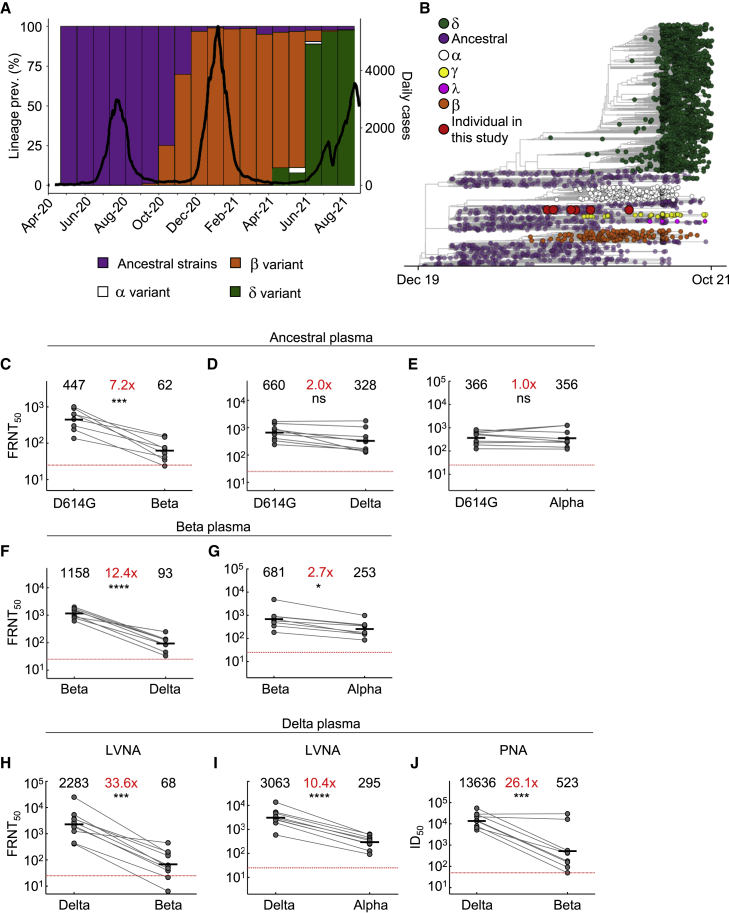
Neutralization distance between variants (A) Infection waves and variant frequencies in South Africa. (B) Maximum-likelihood phylogenetic tree with evolved virus sequences (red) at six time points in relation to 3,883 global sequences with variants shown. (C–E) Neutralization of the Beta (C), Delta (D), and Alpha (E) virus compared to D614G ancestral virus by plasma from convalescent participants infected by ancestral strains (n = 8). (F–G) Neutralization of the Delta (F) and Alpha (G) virus compared to Beta virus by plasma from Beta infections (n = 9). (H–I) Neutralization of the Beta (H) and Alpha (I) viruses compared to Delta virus by plasma from Delta infections (n = 10). Experiments presented in panels C–I performed using a live virus neutralization assay (LVNA). (J) Neutralization of Beta compared to Delta virus with the same plasma as (I) using a pseudo-virus neutralization assay (PNA). Red horizontal line denotes most concentrated plasma tested. Numbers in black above each virus strain are geometric mean titers (GMT) of the reciprocal plasma dilution (FRNT_50_ for LVNA, ID_50_ for PNA) for 50% neutralization. Numbers in red denote fold-change in GMT between virus strain on the left and the virus strain on the right. p values are ^∗^<0.05–0.01, ^∗∗^<0.01–0.001, ^∗∗∗^<0.001–0.0001, and ^∗∗∗∗^<0.0001 as determined by the Wilcoxon rank sum test.

**Figure 2:**
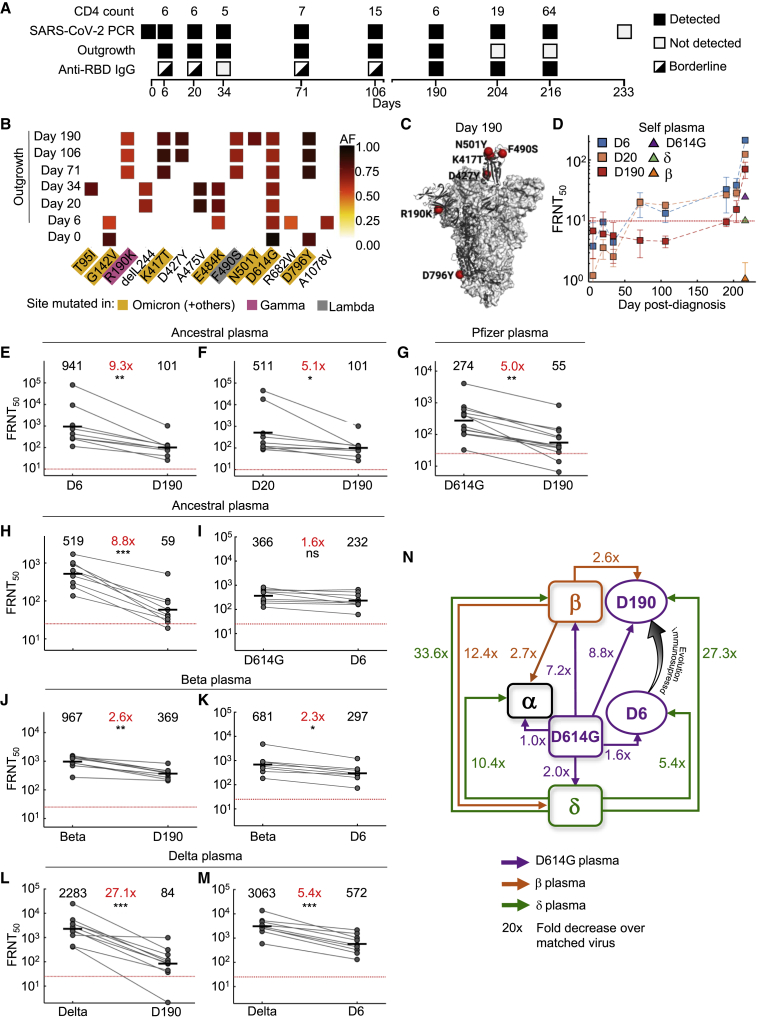
Mapping neutralization of variants and evolved virus (A) Participant characteristics over 233 days from SARS-CoV-2 diagnosis: CD4 T cell count (cells/μL), SARS-CoV-2 detection by qPCR, virus outgrowth success, and presence of anti-RBD IgG. Because IgG levels were close to the background for some time points, they were marked as borderline. (B) Majority and minority SARS-CoV-2 genotypes in the swab (day 0) and outgrowth (day 6 to 190). X axis lists substitutions and deletions in spike sequence, and positions where mutations are found in variants are highlighted. AF: allele frequency. (C) Cryogenic electron microscopy (cryo-EM) structure of the SARS-CoV-2 spike protein. The mutations in day 190 isolated virus (D190) shown as red spheres. (D) Neutralization of day 6 isolated (D6), day 20 isolated (D20), and D190 virus by self-plasma collected days 6 to 216 and the ancestral D614G, Beta, and Delta viruses with plasma collected day 216. (E-F) Neutralization of D6 (E) and D20 (F) relative to D190 virus by ancestral-infection-elicited plasma (n = 8). (G) Neutralization of D190 compared to D614G by Pfizer BNT162b2 plasma (n = 12). (H-I) Neutralization of D190 (H) and D6 (I) compared to D614G by ancestral plasma (n = 8). (J-K) Neutralization of D190 (J) and D6 (K) compared to Beta virus by Beta plasma (n = 9). (L-M) Neutralization of D190 (L) and D6 (M) compared to Delta virus by Delta plasma (n = 10). Red horizontal line denotes most concentrated plasma tested. Numbers in black are GMT FRNT_50_. Numbers in red are fold-change in GMT between virus strain on left and right. p values are ^∗^<0.05–0.01, ^∗∗^<0.01–0.001, and ^∗∗∗^<0.001–0.0001 as determined by the Wilcoxon rank sum test. (N) Summary map (not to scale) of serological distances as measured by fold-decrease in neutralization. For clarity, Beta plasma neutralization of D6 is not shown.

Here we mapped neutralization of ancestral, Beta, Alpha, and Delta variant viruses and a virus evolved in advanced HIV disease immune suppression by antibodies elicited by each strain or variant. These strains were isolated from infections in South Africa. The SARS-CoV-2 antibody neutralization response tested was from the blood of convalescent individuals infected in one of three SARS-CoV-2 infection waves in South Africa, where the first wave was composed of infections by ancestral strains with the D614G substitution, the second dominated by the Beta variant, and the third by the Delta variant (Figure 1A). We obtained viral isolates from the upper respiratory tract and blood-derived plasma from infected individuals in each infection wave, and we sequenced viruses that elicited plasma immunity in order to validate the infecting virus where possible (Table S1 lists study participants related to Figures 1 and 2). A phylogenetic tree of the variants shows the genetic relationships between the Alpha, Beta, Gamma, Delta, and Lambda variants (Figure 1B), as well as the virus evolved from infection of an ancestral SARS-CoV-2 strain in a person with advanced HIV disease (described below).

We used a live virus neutralization assay (LVNA) to quantify neutralization. LVNA reads out as the reduction in the number of infection foci at different neutralizing plasma dilutions, and it is used to obtain the plasma dilution needed for 50% inhibition. We report focus reduction neutralization test 50 (FRNT_50_), the reciprocal of this dilution. We observed that neutralization capacity of ancestral-virus-elicited plasma declined 7.2-fold against the Beta virus (Figure 1C). In contrast, it declined only two-fold against the Delta variant virus (Figure 1D). Neutralization did not decline against the Alpha variant virus (Figure 1E). Using Beta-elicited plasma, we observed a 12.4-fold decline of neutralization of Delta virus relative to Beta virus (Figure 1F). Alpha virus was well neutralized by Beta-elicited plasma, with a 2.7-fold decline compared to Beta virus (Figure 1G). A dramatic decline was observed when Delta-elicited plasma was used to neutralize Beta virus. This resulted in a 33.6-fold drop compared to Delta virus neutralization (Figure 1H). Alpha also showed relatively high neutralization escape from Delta-elicited plasma, at 10.4-fold relative to Delta virus (Figure 1I), although this was considerably lower than Beta escape. Because we have not previously observed the degree of escape in terms of fold-change seen with Beta virus neutralization by Delta-elicited plasma, we repeated the experiments using a pseudovirus neutralization assay (Wibmer et al., 2021). We observed increased sensitivity to neutralization with this system relative to LVNA. However, the drop in neutralization of Beta virus with Delta plasma, at 26.1-fold, was similar to the LVNA results (Figure 1J).

We next characterized SARS-CoV-2 that had evolved in a person with advanced HIV who was diagnosed in late September 2020 with SARS-CoV-2. This person was infected with the ancestral lineage B.1.1.273, which we previously described in a case report (Karim et al., 2021b). The study participant was discharged 9 days post-diagnosis according to South Africa guidelines and remained asymptomatic for most study visits (see description in the Star methods). Due to irregular ART adherence, HIV viremia persisted up to day 190 post-diagnosis. SARS-CoV-2 titer was high throughout this period, ranging from a Ct of 16 to 27 (Karim et al., 2021b). The last positive qPCR result was early May 2021. Phylogenetic mapping is consistent with a single infection event (Figure 1B). The CD4 count was <10 at enrollment (Figure 2A top row). It increased at later time points, possibly due to the improved adherence to ART and a switch to dolutegravir-based therapy which reduced the HIV viral load to below the level of clinical detection (Karim et al., 2021b). SARS-CoV-2 was detected through the use of qPCR until day 216 post-diagnosis (Figure 2A second row). We attempted to isolate live virus up to and including the day 216 post-diagnosis swab sample. Although there was insufficient sample to isolate virus from the day 0 swab, we isolated and expanded SARS-CoV-2 from subsequent swabs up to and including day 190 post-SARS-CoV-2 diagnosis (Figure 2A third row). Successful isolation indicates that live virus was shed at that time. RBD-specific IgG antibodies in the blood were at borderline detection levels (slightly above the mean negative control + 2 std) at the early time points but were detected at higher levels starting day 190 (Figure 2A fourth row, Figure S1 related to Figure 2A).

Outgrown virus was sequenced to detect majority and minority variants (Figure 2B). The mutations found in the outgrown virus were representative of the virus in the swab from the matched time point (Table S2 related to Figure 2 shows read numbers at nucleotides which led to amino acid substitutions or deletions in virus sequenced from the swab and outgrown stock.) except for the R682W substitution at the furin cleavage site on the day 6 sample. This mutation evolves *in vitro* during expansion in Vero E6 cells and likely confers moderate neutralization escape (Johnson et al., 2021). E484K was first detected in the day 6 isolate (Figure 2B). This mutation persisted at days 20 and 34 but was replaced with the F490S substitution starting on day 71, and the K417T mutation was also detected on that day. The N501Y mutation was detected in the virus isolated on day 190 post-diagnosis. Mutations were clustered in the RBD, including K417T, F490S, and N501Y in the day 190 viral isolate (Figure 2C). Among the RBD mutations in the day 190 isolate, K417T is found in the Gamma variant, and F490S is found in the Lambda variant. Among NTD mutations, T95I is found in Mu, and R190K is at the same location as the R190S in Gamma. N501Y is found in Beta, among others. The Omicron variant has emerged as this work was being revised, and it has mutations at many of the same sites as the evolving virus described here (https://covdb.stanford.edu/page/mutation-viewer/#sec_b-1-351). This includes the D796Y mutation which is only found in Omicron among the major variants (Figure 2B).

We tested three of the isolates for neutralization: viruses outgrown from the day 6 and day 20 swabs (designated D6 and D20) representing viruses from early infection, and viruses outgrown from the day 190 swab (D190) after substantial evolution. Neutralization of the D6, D20, and D190 isolates by self-plasma was low at the early time points (Figure 2D). However, neutralization of D6 and D20 was evident in plasma sampled from day 190 and was more pronounced in the plasma sampled from day 216. The D6 isolate was the most sensitive to neutralization by day 216 plasma. Neutralization declined for D20 and further declined for D190, and this result suggests sequential evolution of escape (Figure 2D). The ancestral virus and Beta and Delta variants were also tested for neutralization by using day 216 plasma. Neutralization was lower for all three non-self viral strains relative to self-derived virus. The strongest neutralization was of ancestral virus. Delta was neutralized to a lesser degree, and Beta was not detectably neutralized (Figure 2D).

We also tested the D6, D20, and D190 isolates against plasma from other convalescent participants infected with ancestral virus. Neutralization of D190 by ancestral-infection-elicited plasma was decreased dramatically relative to D6, with FRNT_50_ for D190 being 9.3-fold lower despite the presence of the E484K mutation in D6 (Figure 2E). The difference was smaller between D190 and D20 (5.1-fold, Figure 2F), consistent with evolution of some neutralization escape in D20 relative to D6. We also tested neutralization of D190 virus using Pfizer BNT162b2-vaccinated participants. BNT162b2-elicited plasma neutralization capacity was decreased 5-fold against D190 relative to ancestral virus with the D614G mutation (Figure 2G). We compared neutralization of Beta, D6, D20, and D190 on a subset of remaining BNT162b2 plasma samples from 5 participants 5–6 months post-vaccine, where neutralization declined to relatively low levels. Despite this limitation, neutralization was detectable and showed a pattern consistent with the other results: D190 neutralization escape was very similar to Beta, and D6 and D20 showed no escape from BNT162b2-elicited neutralization (Figure S2 related to Figure 2G). A 5-fold reduction is less than the fold-drop we obtained for the Beta variant with convalescent plasma from previous infection (Cele et al., 2021a), and these results are consistent with substantial but incomplete escape of D190 from BNT162b2-elicited immunity.

We next assessed the serological distance between D190, D6, the ancestral strain, Beta, Delta, and Alpha variants. We tested against convalescent plasma obtained from participants infected with ancestral strains or Beta or Delta variants. Neutralization by ancestral plasma immunity declined 8.8-fold relative to ancestral virus for the D190 isolate (Figure 2H), similar to the Beta variant. In contrast, there was only a 1.6-fold decline for the D6 isolate (Figure 2I). D190 virus was neutralized relatively well by Beta-variant-infection-elicited plasma, with a 2.6-fold reduction (Figure 2J). This was similar to D6 neutralization, which was reduced 2.3-fold relative to Beta (Figure 2K). A much more dramatic decline was observed with Delta-variant-elicited immunity: a 27.1-fold drop in neutralization capacity compared to neutralization of Delta virus (Figure 2L). Escape from Delta-elicited immunity was much more moderate for D6, with a 5.4-fold decline compared to Delta virus (Figure 2M).

Mapping the results (Figure 2N) shows that the Beta and Delta variants are serologically far apart, and ancestral virus forms a hub. The greater distance of Beta relative to Delta from ancestral is consistent with Beta being an escape variant and so evolving antibody escape mutations in the RBD as well as mutations and deletions in the NTD. The Alpha variant was serologically like the ancestral strain and was well neutralized by Beta plasma. However, it did escape Delta-elicited neutralization. D6 was serologically similar to the ancestral strain even though it had the E484K substitution (and the *in vitro*-evolved R682W, which is reported to confer a moderate decrease in neutralization). In contrast, the D190 virus, which underwent extensive evolution, was serologically like Beta despite sequence differences. It had escape from ancestral and a much more pronounced escape from Delta-elicited plasma, similar to the Beta virus. We did not map Gamma, but neutralization escape of the Delta but not the Beta variant from Gamma-elicited neutralization (Liu et al., 2021) may suggest that Beta and Gamma are serologically similar.

We have shown that Delta, Beta, Alpha, and the D190 virus we characterize here are all escape variants and/or strains in the sense that each can escape from neutralizing immunity elicited by at least one other variant. This is true even though the D190 virus and Beta and Alpha variants predate Delta, and so escape from Delta immunity could not have been selected for. Furthermore, Delta shows weak escape and Alpha shows no escape from immunity elicited by ancestral strains, and this indicates that they may have evolved to better transmit and not to escape.

Escape between variants which was not selected for could happen because antibodies are elicited to preferred sites on spike, and these differ between variants. Antibodies against the RBD of ancestral strains are concentrated around the E484 site (class 2) which differs between ancestral and Beta (Greaney et al., 2021a). Therefore, cross-neutralization of Beta virus by ancestral-infection-elicited immunity is weak. In contrast, Beta elicits a stronger response to the class 3 epitope spanning sites 443 to 452 in the RBD (Greaney et al., 2021c), where Beta and ancestral do not differ. Therefore, cross-neutralization of ancestral virus by Beta immunity is effective. Delta and Beta do differ at class 3 sites, possibly leading to Delta escape of Beta immunity and vice versa. Differences in the NTD would further decrease cross-neutralization.

The driving force behind the evolution of D190 may have been the presence of very low levels of antibody to SARS-CoV-2, which may select for antibody escape mutations without the ability to clear the infection. Although the participant described here did develop a neutralization response and did clear the virus, the response was strongest against the ancestral-like virus early in infection and weak for the Beta-like D190 isolate, and this indicates a considerable lag between SARS-CoV-2 evolution and neutralization. Consistent with this, neutralization was detectable for the ancestral strain but not the Beta virus. This may be a source of vulnerability for future SARS-CoV-2 infections in the participant.

Antigenic cartography has been extensively used in influenza (Smith et al., 2004). Genetic distance may be partially but not completely a proxy for antigenic distance because some substitutions can lead to large changes in antigenicity while others lead to minor effects (Smith et al., 2004). It is unclear whether genetic distance is a good measure for antigenic differences between SARS-CoV-2 variants. For example, based on the phylogenetic tree (Figure 1B), it is not obvious that Beta-elicited immunity would neutralize Alpha virus better than Delta immunity would. Perhaps this is because SARS-CoV-2 evolution seems be closer to that of HIV, in which multiple strains radiate from a common ancestor (Korber et al., 2000), than to the stepwise progression in influenza (Smith et al., 2004). How far the variants will diverge from each other is unclear, but if further divergence occurs, SARS-CoV-2 may form serotypes such as those that occur with polio (Minor et al., 1986) and dengue (Guzman et al., 2007) viruses. With serotypes, antibody immunity is developed to the infecting serotype but not the other serotypes. This may imply that a vaccine which is based on one variant or strain or previous infection with that variant may generate immunity which is vulnerable to infection by another variant.

What surprised us in the participant from whom we derived the viral isolates was that, over the 6-month period in which SARS-CoV-2 titer was high, infection was for the most part asymptomatic. One explanation is that the virus evolved attenuated pathogenicity or had low pathogenicity to begin with. This would account for the mild disease which allowed the individual harboring the infection to survive and the virus to evolve. Another explanation may be that high pathogen levels are tolerated because immunosuppression reduces the inflammatory response. This latter scenario is observed in tuberculosis (Meintjes et al., 2018).

While this work was being revised, the Omicron variant has been detected in South Africa and has spread globally. Omicron (B.1.1.529) is derived from strains which were common in the first infection wave in South Africa, 1.5 years ago, but were then supplanted first by Beta, then Delta. There may be several possibilities for how this virus persisted so long without being detected. One explanation is that Omicron may have evolved in a region with less developed genomic surveillance relative to South Africa and may have arrived in South Africa recently. Alternatively and without excluding the former possibility, it may have persisted in a single immune-compromised host until it acquired a critical mass of mutations that would allow it to effectively transmit in a population where the prevalence of previous infection is high (Khan et al., 2021, Sykes et al., 2021). In support of this, previous studies that investigated SARS-CoV-2 evolution in people immune-compromised for reasons other than advanced HIV disease have established that such evolution leads to immune escape mutations (Chen et al., 2021, Clark et al., 2021). Therefore, evolution of escape is not related directly to HIV but rather to severe damage to the immune response from long-term, poorly suppressed HIV infection. If the evolved viral strain described in this work and Omicron share a common evolutionary mechanism, other features could also be shared, such as potentially mild pathogenicity (Goga et al., 2021, Jassat et al., 2021) which could account for long-term persistence in a single host.

## Limitations of the study

A limitation of this study is that we have characterized one case. Out of 93 people living with HIV (PLWH) in our cohort at the time of analysis (Karim et al., 2021a), 13 had persistent CD4 < 200 but only one (∼1%) showed extensive evolution of SARS-CoV-2 as described here. However, as there are about 8 million PLWH in South Africa (https://www.unaids.org/en/regionscountries/countries/southafrica), assuming a frequency of 1% would translate to 80,000 people where SARS-CoV-2 evolution could occur. We have previously reported on a 2-fold drop in the number of SARS-CoV-2 convalescents suppressed with antiretroviral therapy in the second versus the first infection wave in South Africa (Karim et al., 2021a). If immunosuppression by advanced HIV drives SARS-CoV-2 evolution, ART coverage should be increased to prevent it.

## Consortia

Members of the COMMIT-KZN Team: Moherndran Archary, Philip Goulder, Nokwanda Gumede, Ravindra K. Gupta, Guy Harling, Rohen Harrichandparsad, Kobus Herbst, Prakash Jeena, Zesuliwe Jule, Thandeka Khoza, Nigel Klein, Henrik Kloverpris, Alasdair Leslie, Rajhmun Madansein, Mohlopheni Marakalala, Yoliswa Miya, Mosa Moshabela, Nokukhanya Msomi, Kogie Naidoo, Zaza Ndhlovu, Kennedy Nyamande, Vinod Patel, Dirhona Ramjit, Kajal Reedoy, Theresa Smit, Adrie Steyn, and Emily Wong

## STAR★Methods

### Key resources table

**Table:** 

Reagent or resource	Source	Identifier
**Antibodies**		

MonoRab™ SARS-CoV-2 Neutralizing Antibody (BS-R2B2), mAb, Rabbit	GenScript	GenScript Cat#: A02051
MonoRab™ SARS-CoV-2 Spike S1 Antibody (BS-R2B12), mAb, Rabbit	GenScript	GenScript Cat #:A02058
Goat Anti-Rabbit IgG H&L (HRP) for LVNA	Abcam	ab205718; RRID:AB_2819160
Goat anti-human IgG (HRP) for ELISA	Jackson ImmunoResearch	709-036-098; RRID:AB_2340497
SARS-CoV-2 Spike S1 Antibody (HC2001), Human Chimeric	Dr Galit Alter, Ragon Institute, USA. Also available from GenScript	GenScript Cat#: A02038
CB6 plasmid—used to express the CB6 antibody in house.	GenScript	Custom synthesized

**Bacterial and virus strains**		

B.1.1.117 (ancestral SARS-CoV-2)	Cele et al., 2021a	EPI_ISL_602622 (GISAID accession)
B.1.351 (Beta variant)	Cele et al., 2021a	EPI_ISL_678615 (GISAID accession)
B.1.617.2 (Delta variant)	South Africa cohort described here.	EPI_ISL_3118687 (GISAID accession)
B.1.1.7 (Alpha variant)	KwaZulu-Natal Research Innovation and Sequencing Platform	EPI_ISL_2086212 (GISAID accession)
Adv. HIV disease SARS-CoV-2 day 0 isolate	This paper	EPI_ISL_602912 (GISAID accession)
Adv. HIV disease SARS-CoV-2 day 6 isolate	This paper	EPI_ISL_2397308 (GISAID accession)
Adv. HIV disease SARS-CoV-2 day 20 isolate	This paper	EPI_ISL_2397310 (GISAID accession)
Adv. HIV disease SARS-CoV-2 day 34 isolate	This paper	EPI_ISL_2397311 (GISAID accession)
Adv. HIV disease SARS-CoV-2 day 71 isolate	This paper	EPI_ISL_2397312 (GISAID accession)
Adv. HIV disease SARS-CoV-2 day 106 isolate	This paper	EPI_ISL_2397309 (GISAID accession)
Adv. HIV disease SARS-CoV-2 day 190 isolate	This paper	EPI_ISL_2397313 (GISAID accession)

**Biological samples**		

Samples from SARS-CoV-2 convalescent participants	South Africa cohort described here.	Described Table S1 and Ethical Statement
Samples from Pfizer BNT162b2-vaccinated participants	US cohort described here.	Described Table S4 and Ethical Statement

**Chemicals, peptides, and recombinant proteins**		

Carboxymethylcellulose	SIGMA	Cat#C4888
TrueBlue peroxidase substrate	SeraCare	Cat#5510-0030
SARS-CoV-2 Spike protein (RBD, His Tag)	Galit Alter, Ragon Institute, USA. Also available from GenScript	Z03479 (Genscript)
Ultra TMB substrate	ThermoFisher	Cat#34028
RPMI-1640, powder	SIGMA	Cat#R6504
Saponin	SIGMA	Cat#S7900

**Critical commercial assays**		

Superscript IV First Strand synthesis system	Life Technologies	Cat#18091050
AmpureXP purification beads	Beckman Coulter	Cat#A63880
Qubit dsDNA High Sensitivity assay	ThermoFisher	Cat#Q32851
Illumina Nextera Flex DNA Library Prep kit	Illumina	Cat#20018705
PhiX Control v3	Illumina	Cat#FC-110-3001
MiSeq Reagent Kit v2 (500-cycles)	Illumina	Cat#MS-102-2003

**Deposited data**		

B.1.617.2 (Delta variant)	GISAID	EPI_ISL_3118687
B.1.1.7 (Alpha variant)	GISAID	EPI_ISL_2086212
Adv. HIV disease SARS-CoV-2 day 0 isolate	GISAID	EPI_ISL_602912
Adv. HIV disease SARS-CoV-2 day 6 isolate	GISAID	EPI_ISL_2397308
Adv. HIV disease SARS-CoV-2 day 20 isolate	GISAID	EPI_ISL_2397310
Adv. HIV disease SARS-CoV-2 day 34 isolate	GISAID	EPI_ISL_2397311
Adv. HIV disease SARS-CoV-2 day 71 isolate	GISAID	EPI_ISL_2397312
Adv. HIV disease SARS-CoV-2 day 106 isolate	GISAID	EPI_ISL_2397309
Adv. HIV disease SARS-CoV-2 day 190 isolate	GISAID	EPI_ISL_2397313

**Experimental models: Cell lines**		

Vero E6	ATCC (obtained from Cellonex in South Africa)	CRL-1586 (ATCC)
H1299-ACE2 clone H1299-E3	Cele et al., 2021a	N/A
HEK293T-ACE2	Dr Michael Farzan, Scripps, USA	N/A

**Software and algorithms**		

ARTIC V.3 protocol	ARTIC Network	https://www.protocols.io/view/covid-19-artic-v3-illumina-library-construction-an-bibtkann
MATLAB v.2019b	Mathworks	https://www.mathworks.com/products/matlab.html
Primal Scheme	Quick J et al., 2017	http://primal.zibraproject.org/
Genome Detective 1.126 – Coronavirus Typing Tool	(Vilsker et al., 2019)	https://www.genomedetective.com
bcftools 1.7-2 mpileup	(Li et al., 2009)	https://samtools.github.io/bcftools/bcftools.html
Geneious	Biomatters	https://www.geneious.com/
SARS-CoV-2 NextStrain	https://nextstrain.org/ncov/gisaid/global	https://github.com/nextstrain/ncov
ggTree	Yu G et al., 2020	https://bioconductor.org/packages/release/bioc/html/ggtree.html
ggPlot	Wickham H et al., 2016	https://ggplot2.tidyverse.org/

### Resource availability

#### Lead contact

Further information and requests for reagents may be directed to and will be fulfilled by lead contact, Alex Sigal (alex.sigal@ahri.org)

#### Materials availability

SARS-CoV-2 isolates used in this study are available from the lead contact upon request.

### Experimental model and subject details

#### Ethical statement

Nasopharyngeal and oropharyngeal swab samples and blood samples were obtained after written informed consent from hospitalized adults with PCR-confirmed SARS-CoV-2 infection or vaccinated individuals who were enrolled in a prospective cohort study approved by the Biomedical Research Ethics Committee at the University of KwaZulu–Natal (reference BREC/00001275/2020). The participant with advanced HIV disease described in this report was enrolled under the same study (reference BREC/00001275/2020). We obtained additional written informed consent for publication from the participant with advanced HIV disease. The Pfizer BNT162b2 plasma was acquired from participants enrolled in the UWARN: COVID-19 in WA study (STUDY00010350) approved by the University of Washington Human Subjects Division IRB.

#### Participants

Participant with advanced HIV disease was a female in her late 30 s symptomatic for Covid-19 admitted to a hospital in Durban, South Africa. Symptom onset was 12 days prior to hospital admission. Participant was admitted to hospital in September 2020 with shortness of breath, sore throat, and cough and administered non-high flow supplemental oxygen via face mask and a six-day course of dexamethasone. Study enrollment was in October 2020, 6 days post-admission, and each study visit consisted of a blood draw and a combined nasopharyngeal and oropharyngeal swab to detect and isolate SARS-CoV-2. Study visits occurred at enrollment, and day 20, 34, 71, 106, 190, 204, 216, and 233 post-admission. Participant had been on ART since 2006, most recently a fixed-dose combination of tenofovir, emtricitabine and efavirenz. However, ART components were not detected by liquid chromatography-tandem mass spectrometry at enrollment. Participant was discharged 9 days post-admission. On day 20 and day 34 post-admission, participant was asymptomatic. On day 71, she complained of chest tightness and oxygen saturation decreased from 96% to 76% on exertion. Chest X-ray showed perihilar infiltrates and participant was treated for *Pneumocystis* pneumonia (PCP) as an outpatient with 21 days of co-trimoxazole and prednisone. She was reviewed on day 106 post-admission when she reported fatigue, and on day 190 when she was asymptomatic. On day 190 post-admission, ART was switched to a fixed-dose combination of tenofovir, lamivudine and dolutegravir. HIV viral load was suppressed on day 204 post-admission. No symptoms were reported on day 190, 204, 216 or 233 post-admission. More information can be found in the case report (Karim et al., 2021b). Characteristics of convalescent participants with ancestral, Beta, or Delta variant infection whose blood was used for neutralization experiments can be found in Table S1, and Pfizer BNT162b2-vaccinated blood donor characteristics can be found in Table S4.

#### Cells

Vero E6 cells (ATCC CRL-1586, obtained from Cellonex in South Africa) were propagated in complete DMEM with 10% fetal bovine serum (Hylone) containing 1% each of HEPES, sodium pyruvate, L-glutamine and nonessential amino acids (Sigma-Aldrich). Vero E6 cells were passaged every 3–4 days. The H1299-E3 cell line for first-passage SARS-CoV-2 expansion, derived as described in (Cele et al., 2021a), was propagated in complete RPMI with 10% fetal bovine serum containing 1% each of HEPES, sodium pyruvate, L-glutamine and nonessential amino acids. H1299 cells were passaged every second day. Cell lines have not been authenticated. The cell lines have been tested for mycoplasma contamination and are mycoplasma negative.

#### Virus expansion

All work with live virus was performed in Biosafety Level 3 containment using protocols for SARS-CoV-2 approved by the AHRI Biosafety Committee. We used ACE2-expressing H1299-E3 cells for the initial isolation (P1 stock) followed by passaging in Vero E6 cells (P2 and P3 stocks, where P3 stock was used in experiments). ACE2-expressing H1299-E3 cells were seeded at 4.5 × 10^5^ cells in a 6 well plate well and incubated for 18–20 h. After one DPBS wash, the sub-confluent cell monolayer was inoculated with 500 μL universal transport medium diluted 1:1 with growth medium filtered through a 0.45-μm filter. Cells were incubated for 1 h. Wells were then filled with 3 mL complete growth medium. After 8 days of infection, cells were trypsinized, centrifuged at 300 rcf for 3 min and resuspended in 4 mL growth medium. Then 1 mL was added to Vero E6 cells that had been seeded at 2 × 10^5^ cells per mL 18–20 h earlier in a T25 flask (approximately 1:8 donor-to-target cell dilution ratio) for cell-to-cell infection. The coculture of ACE2-expressing H1299-E3 and Vero E6 cells was incubated for 1 h and the flask was then filled with 7 mL of complete growth medium and incubated for 6 days. The viral supernatant (P2 stock) was aliquoted and stored at −80 °C and further passaged in Vero E6 cells to obtain the P3 stock used in experiments.

### Method details

#### Whole-genome sequencing, genome assembly, and phylogenetic analysis

cDNA synthesis was performed on the extracted RNA using random primers followed by gene-specific multiplex PCR using the ARTIC V.3 protocol (https://www.protocols.io/view/covid-19-artic-v3-illumina-library-construction-an-bibtkann). In brief, extracted RNA was converted to cDNA using the Superscript IV First Strand synthesis system (Life Technologies) and random hexamer primers. SARS-CoV-2 whole-genome amplification was performed by multiplex PCR using primers designed using Primal Scheme (http://primal.zibraproject.org/) to generate 400-bp amplicons with an overlap of 70 bp that covers the 30 kb SARS-CoV-2 genome. PCR products were cleaned up using AmpureXP purification beads (Beckman Coulter) and quantified using the Qubit dsDNA High Sensitivity assay on the Qubit 4.0 instrument (Life Technologies). We then used the Illumina Nextera Flex DNA Library Prep kit according to the manufacturer’s protocol to prepare indexed paired-end libraries of genomic DNA. Sequencing libraries were normalized to 4 nM, pooled and denatured with 0.2 N sodium acetate. Then, a 12-pM sample library was spiked with 1% PhiX (a PhiX Control v.3 adaptor-ligated library was used as a control). We sequenced libraries on a 500-cycle v.2 MiSeq Reagent Kit on the Illumina MiSeq instrument (Illumina). We assembled paired-end fastq reads using Genome Detective 1.126 (https://www.genomedetective.com) and the Coronavirus Typing Tool. We polished the initial assembly obtained from Genome Detective by aligning mapped reads to the reference sequences and filtering out low-quality mutations using the bcftools 1.7-2 mpileup method. Mutations were confirmed visually with BAM files using Geneious software (Biomatters). We analyzed sequences from the six different time points (D6, D20, D34, D71, D106 and D190) against a global reference dataset of 3883 genomes using a custom build of the SARS-CoV-2 NextStrain (https://github.com/nextstrain/ncov). The workflow performs alignment of genomes, phylogenetic tree inference, tree dating and ancestral state construction and annotation. The phylogenetic tree was visualized using ggplot and ggtree. All sequences from the participant clustered in a monophyletic clade (https://nextstrain.org/groups/ngs-sa/COVID19-AHRI-2021.05.27?label=clade:HIV%20patient) that are well separated from the rest of the phylogeny.

#### Live virus neutralization assay

Vero E6 cells were plated in a 96-well plate (Corning) at 30,000 cells per well 1 day pre-infection. Plasma was separated from EDTA-anticoagulated blood by centrifugation at 500 rcf for 10 min and stored at −80 °C. Aliquots of plasma samples were heat-inactivated at 56 °C for 30 min and clarified by centrifugation at 10,000 rcf for 5 min. GenScript A02051 anti-spike neutralizing monoclonal antibody was added as a positive control to one column of wells. Virus stocks were used at approximately 50-100 focus-forming units per microwell and added to diluted plasma. Antibody–virus mixtures were incubated for 1 h at 37 °C, 5% CO_2_. Cells were infected with 100 μL of the virus–antibody mixtures for 1 h, then 100 μL of a 1X RPMI 1640 (Sigma-Aldrich, R6504), 1.5% carboxymethylcellulose (Sigma-Aldrich, C4888) overlay was added without removing the inoculum. Cells were fixed 18 h post-infection using 4% PFA (Sigma-Aldrich) for 20 min. Foci were stained with a rabbit anti-spike monoclonal antibody (BS-R2B12, GenScript A02058) at 0.5 μg/mL in a permeabilization buffer containing 0.1% saponin (Sigma-Aldrich), 0.1% BSA (Sigma-Aldrich) and 0.05% Tween-20 (Sigma-Aldrich) in PBS. Plates were incubated with primary antibody overnight at 4 °C, then washed with wash buffer containing 0.05% Tween-20 in PBS. Secondary goat anti-rabbit horseradish peroxidase (Abcam ab205718) antibody was added at 1 μg/mL and incubated for 2 h at room temperature with shaking. TrueBlue peroxidase substrate (SeraCare 5510-0030) was then added at 50 μL per well and incubated for 20 min at room temperature. Plates were imaged in an ELISPOT instrument with built-in image analysis (C.T.L).

#### Pseudovirus neutralization assay

SARS-CoV-2 pseudotyped lentiviruses were prepared by co-transfecting the HEK293T cell line with either the SARS-CoV-2 Beta spike (L18F, D80A, D215G, K417N, E484K, N501Y, D614G, A701V, 242-244 del) or the Delta spike (T19R, R158G L452R, T478K, D614G, P681R, D950N, 156-157 del) plasmids in conjunction with a firefly luciferase encoding lentivirus backbone plasmid. For the neutralization assay, heat-inactivated plasma samples from vaccine recipients were incubated with the SARS-CoV-2 spike pseudotyped virus for 1 h at 37°C, 5% CO_2_. Subsequently, 1x10^4^ HEK293T cells engineered to overexpress ACE-2 were added and incubated at 37°C, 5% CO_2_ for 72 h upon which the luminescence of the luciferase gene was measured. CB6 antibody was used as a positive control.

#### Receptor binding domain ELISA

Plasma samples were tested for anti-SARS-CoV-2 IgG. Flat bottom microplates (ThermoFisher Scientific) were coated with 500 ng/mL of the receptor-binding domain (RBD) protein (provided by Dr Galit Alter from the Ragon Institute) and incubated overnight at 4°C. Plates were blocked with a 200 μL/well tris-buffered saline containing 1% BSA (TBSA) and incubated at room temperature (RT) for 1 h. Samples were diluted in TBSA with 0.05% Tween-20 (TBSAT) to 1:100. Subsequently, goat anti-human IgG (1:5000)- horseradish peroxidase conjugated secondary antibodies (Jackson ImmunoResearch) were added a 100 μL/well and incubated at RT for 1 h. Bound secondary antibodies were detected using 100 μL/well 1-step Ultra TMB substrate (ThermoFisher Scientific). Plates were incubated at RT for 3 min in the dark before addition of 1 N sulphuric acid stop solution at 100 μL/well. Plates were washed with 1X high salt TBS containing 0.05% Tween-20, three times each after coating and blocking, and five times each after the sample and secondary antibody. The concentration of anti-RBD expressed as ng/mL equivalent of anti-SARS-CoV-2 monoclonal, CR3022 (Genscript). We used pre-pandemic plasma samples as negative controls to define seroconversion cut-offs calculated as mean + 2 std of the negative samples.

### Quantification and statistical analysis

Details of all statistical analyses used in this study as well as the number of samples tested are described in detail in the respective figure legends. All statistics and fitting were performed using MATLAB v.2019b. Neutralization data were fit to:

Tx = 1/1+(D/ID50).

Here Tx is the number of foci normalized to the number of foci in the absence of plasma on the same plate at dilution D and ID_50_ is the plasma dilution giving 50% neutralization. FRNT_50_ = 1/ID50. Values of FRNT_50_ < 1 are set to 1 (undiluted), the lowest measurable value.

## Data Availability

• Sequence of outgrown virus has been deposited in GISAID. Raw images of the data are available upon reasonable request. • Image analysis and curve fitting scripts in MATLAB v.2019b are available on GitHub (https://github.com/sigallab/NatureMarch2021). • Any additional information required to reanalyze the data reported in this paper is available from the lead contact upon request except for identifying participant information.

## Supplemental information

Document S1. Tables S1 and S2 and Figures S1 and S2

Document S2. Article plus supplemental information
