# Supplementary material for: SARS-CoV-2 prolonged infection during advanced HIV disease evolves extensive immune escape
Source: Cell Host Microbe. 2022 Feb 9;30(2):154–162.e5. doi: 10.1016/j.chom.2022.01.005 (PMC8758318; doi:10.1016/j.chom.2022.01.005)
Supplement: Document S1. Tables S1 and S2 and Figures S1 and S2 [file mmc1.pdf]

Cell Host & Microbe, Volume *30*

Supplemental information

SARS-CoV-2 prolonged infection during advanced HIV disease evolves extensive immune escape

Sandile Cele, Farina Karim, Gila Lustig, James Emmanuel San, Tandile Hermanus, Houriiyah Tegally, Jumari Snyman, Thandeka Moyo-Gwete, Eduan Wilkinson, Mallory Bernstein, Khadija Khan, Shi-Hsia Hwa, Sasha W. Tilles, Lavanya Singh, Jennifer Giandhari, Ntombifuthi Mthabela, Matilda Mazibuko, Yashica Ganga, Bernadett I. Gosnell, Salim S. Abdool Karim, Willem Hanekom, Wesley C. Van Voorhis, Thumbi Ndung'u, COMMIT-KZN Team, Richard J. Lessells, Penny L. Moore, Mahomed-Yunus S. Moosa, Tulio de Oliveira, and Alex Sigal

Supplementary materials for SARS-CoV-2 prolonged infection during advanced HIV disease evolves extensive immune escape

Cele et al.

Contents

1 **Table S1: Characteristics of SARS-CoV-2 convalescent or vaccinated study participants related to Figures 1 and 2** **2**

2 **Table S2: Read counts of majority and minority genotypes detected in swab and outgrown virus related to Figure 2** **3**

3 **Figure S1: Spike specific antibody levels with time post-SARS-CoV-2 diagnosis related to Figure 2.** **4**

4 **Figure S2: Neutralization of Beta variant and evolved virus by a subset of BNT162b2 plasma donors related to Figure 2** **4**

1 Table S1: Characteristics of SARS-CoV-2 convalescent or vaccinated study participants related to Figures 1 and 2

Participant number	Infected or BNT162b2 vaccinated	Variant/strain by infection date	Variant/strain by sequencing	Sequence ID	GISAID Accession	Age range (y)	Sex	Symptom onset or vaccination to plasma collection (days)
**1**	Infected	Ancestral	B.1.1.273	K008646	EPI_ISL_2397308	30-39	F	6, 20, 26, 34, 71, 106, 190
**2**	Infected	Ancestral	B.1.1	K003667	EPI_ISL_602623	50-59	M	29
**3**	Infected	Ancestral	B.1.1.273	K003675	EPI_ISL_602631	40-49	F	32
**4**	Infected	Ancestral	B.1.1.117	K003668	EPI_ISL_602624	70+	F	29
**5**	Infected	Ancestral	B.1.1	K004289	EPI_ISL_660170	60-69	F	27
**6**	Infected	Ancestral	B.1.140	K004295	EPI_ISL_660176	60-69	F	28
**7**	Infected	Ancestral	B.1.1.84	K003673	EPI_ISL_602629	40-49	F	28
**8**	Infected	Ancestral	B.1	K004291	EPI_ISL_660172	30-39	F	30
**9**	Infected	Ancestral	C.9	K004302	EPI_ISL_660181	60-69	M	26
**10**	Infected	Beta	Beta	K008635	N/A*	40-49	F	30
**11**	Infected	Beta	Beta	K008636	N/A*	40-49	F	41
**12**	Infected	Beta	Beta	K008628	N/A*	40-49	M	32
**13**	Infected	Beta	Beta	K008637	EPI_ISL_1229368	50-59	M	42
**14**	Infected	Beta	N/A$	N/A$	N/A	30-39	F	32
**15**	Infected	Beta	Beta	K010372	N/A*	30-39	M	33
**16**	Infected	Beta	Beta	K010356	N/A*	70+	F	48
**17**	Infected	Beta	Beta	K008633	EPI_ISL_1229367	60-69	F	29
**18**	Infected	Beta	Beta	K010370	N/A*	30-39	F	31
**19**	Infected	Delta	Delta	K021407	EPI_ISL_3722338	40-49	F	26
**20**	Infected	Delta	Delta	K021399	EPI_ISL_3722335	40-49	M	23#
**21**	Infected	Delta	Delta	K021401	N/A*	50-59	M	31
**22**	Infected	Delta	Delta	K021225	N/A*	40-49	M	13#
**23**	Infected	Delta	Delta	K021400	N/A*	40-49	M	44
**24**	Infected	Delta	Delta	K021226	N/A*	50-59	M	44
**25**	Infected	Delta	Delta	K020186	EPI_ISL_3939068	40-49	M	31
**26**	Infected	Delta	Delta	K020187	EPI_ISL_3939088	30-39	M	31
**27**	Infected	Delta	Delta	K020214	EPI_ISL_3447779	50-59	F	30#
**28**	Infected	Delta	Delta	K021404	N/A*	30-39	M	32
**29**	BNT162b2	-	-	-	-	60-69	M	11
**30**	BNT162b2	-	-	-	-	60-69	M	10
**31**	BNT162b2	-	-	-	-	50-59	M	18
**32**	BNT162b2	-	-	-	-	40-49	F	9
**33**	BNT162b2	-	-	-	-	60-69	M	10
**34**	BNT162b2	-	-	-	-	20-29	F	8
**35**	BNT162b2	-	-	-	-	60-69	M	128
**36**	BNT162b2	-	-	-	-	30-39	F	134
**37**	BNT162b2	-	-	-	-	20-29	M	131
**38**	BNT162b2	-	-	-	-	50-59	M	152
**39**	BNT162b2	-	-	-	-	30-39	F	153
**40**	BNT162b2	-	-	-	-	30-39	M	158

N/A – Not available. * <90% coverage. Not submitted to GISAID but sufficient sequence for variant call. $ Not sequenced. #Asymptomatic at diagnosis, date post-diagnostic test used instead of symptom onset date.

2 Table S2: Read counts of majority and minority genotypes detected in swab and outgrown virus related to Figure 2

AA change	Nucleotide change	Codon change	Day 0	Day 6		Day 20		Day 34		Day 71		Day 106		Day 190	
			Swab	Swab	Outgrowth	Swab	Outgrowth	Swab	Outgrowth	Swab	Outgrowth	Swab	Outgrowth	Swab	Outgrowth
T95I	21846C>T	21845<br>ACT > ATT	ACT – 143<br>ATT – 0	ACT – 6105<br>ATT - 9	ACT – 1342<br>ATT - 10	ACT – 2667<br>ATT - 298	ACT – 1540<br>ATT - 33	ACT – 935<br>ATT – 1626	ACT – 96<br>ATT – 1328	ACT – 1379<br>ATT – 36	ACT – 1609<br>ATT – 27	ACT –11843<br>ATT – 576	ACT – 1593<br>ATT – 29	ACT – 231<br>ATT – 10	ACT – 1325<br>ATT – 16
G142V	21987G>T	21986<br>GGT>GTT	GGT – 10<br>GTT – 40	GGT – 1386<br>GTT - 622	GGT – 316<br>GTT - 808	GGT – 880<br>GTT - 313	GGT – 1352<br>GTT - 4	GGT – 954<br>GTT – 96	GGT – 1238<br>GTT – 14	GGT – 480<br>GTT – 5	GGT – 1416<br>GTT – 2	GGT – 3621<br>GTT – 12	GGT – 1400<br>GTT – 5	GGT – 133<br>GTT – 22	GGT – 977<br>GTT – 6
R190K	22131G>A	22130<br>AGG>AAG	AGG – 46<br>AAG – 0	AGG – 5591<br>AAG – 8	AGG – 119<br>AAG – 0	AGG – 1702<br>AAG – 1	AGG – 357<br>AAG – 3	AGG –1685<br>AAG - 2	AGG – 324<br>AAG - 2	AGG – 222<br>AAG – 1096	AGG – 26<br>AAG – 378	Gap in<br>sequence	AGG – 22<br>AAG – 468	AGG – 13<br>AAG – 40	AGG – 419<br>AAG – 33
*L244del	22293<br>ACA>del	22811<br>ACA>del	ACA – 12<br>del – 1	ACA – 1378<br>del - 0	ACA – 371<br>del - 4	ACA – 50<br>del – 158	ACA – 0<br>del – 635	ACA – 17<br>del – 176	ACA – 17<br>del – 556	ACA – 414<br>del – 4	ACA – 692<br>del – 3	ACA – 11<br>del – 0	ACA – 693<br>del – 7	ACA – 64<br>del – 0	ACA – 653<br>del – 1
K417T	22812A>G	22811<br>AAG>ACG	AAG – 117<br>ACG – 0	AAG – 3237<br>ACG - 1	AAG – 141<br>ACG - 0	AAG – 3209<br>ACG – 0	AAG – 122<br>ACG – 0	AAG – 2587<br>ACG – 1	AAG – 96<br>ACG – 0	AAG – 299<br>ACG – 1362	AAG – 6<br>ACG – 172	AAG – 32<br>ACG – 2	AAG – 0<br>ACG – 60	Gap in<br>sequence	AAG – 0<br>ACG – 17
D427Y	22841G>T	22925<br>GAT>TAT	GAT – 107<br>TAT – 0	GAT – 3856<br>TAT – 3	GAT – 1281<br>TAT – 7	GAT – 3719<br>TAT – 0	GAT – 1273<br>TAT – 1	GAT – 3434<br>TAT – 0	GAT – 1164<br>TAT - 1	GAT – 3434<br>TAT – 0	GAT – 1079<br>TAT – 42	GAT – 51<br>TAT – 4	GAT – 13<br>TAT – 743	GAT – 18<br>TAT - 103	GAT – 6<br>TAT - 529
L455F	22927G>T	22925<br>TTG>TTT	TTG – 11<br>TTT – 0	TTG – 3714<br>TTT – 9	TTG – 886<br>TTT – 0	TTG – 3219<br>TTT – 0	TTG – 978<br>TTT – 2	TTG – 4331<br>TTT – 2	TTG – 888<br>TTT – 3	TTG –2983<br>TTT – 4	TTG – 784<br>TTT – 2	TTG –527<br>TTT – 317	TTG –636<br>TTT – 0	TTG – 114<br>TTT – 0	TTG – 454<br>TTT – 1
F456L	22928T>C	22928<br>TTT>CTT	TTT – 12<br>CTT – 0	TTT – 3739<br>CTT – 6	TTT – 1112<br>CTT – 31	TTT – 3248<br>CTT – 0	TTT – 1109<br>CTT – 19	TTT – 4361<br>CTT – 0	TTT – 1011<br>CTT – 20	TTT – 2971<br>CTT – 3	TTT – 882<br>CTT – 19	TTT – 537<br>CTT – 310	TTT – 725<br>CTT – 2	TTT – 138<br>CTT – 0	TTT – 544<br>CTT – 24
A475V	22986C>T	22985<br>GCC>GTC	GCC – 17<br>GTC – 0	GCC – 3409<br>GTC - 11	GCC – 1180<br>GTC - 30	GCC – 3629<br>GTC – 1218	GCC – 11<br>GTC – 1369	GCC – 2874<br>GTC – 2421	GCC – 43<br>GTC – 1224	GCC – 3396<br>GTC – 101	GCC – 956<br>GTC – 22	GCC – 901<br>GTC – 23	GCC – 839<br>GTC – 24	GCC – 177<br>GTC – 14	GCC – 687<br>GTC – 19
E484K	23012G>A	23012<br>GAA>AAA	GAA – 17<br>AAA – 0	GAA – 56<br>AAA - 2063	GAA – 363<br>AAA - 917	GAA – 1632<br>AAA – 2881	GAA – 27<br>AAA – 1276	GAA – 2395<br>AAA – 2139	GAA – 55<br>AAA – 1133	GAA – 3147<br>AAA – 196	GAA – 1120<br>AAA – 22	GAA – 688<br>AAA – 40	GAA – 1001<br>AAA – 5	GAA – 180<br>AAA – 53	GAA – 814<br>AAA – 4
F490S	23031T>C	23030<br>TTT>TCT	TTT – 18<br>TCT – 0	TTT- 1605<br>TCT - 2	TTT- 1196<br>TCT - 15	TTT – 4067<br>TCT – 0	TTT – 1226<br>TCT – 10	TTT – 4007<br>TCT – 8	TTT – 1031<br>TCT – 30	TTT – 341<br>TCT – 2912	TTT – 62<br>TCT – 935	TTT – 182<br>TCT – 446	TTT – 31<br>TCT – 784	TTT – 89<br>TCT – 125	TTT – 21<br>TCT – 669
N501Y	23063A>T	23063<br>AAT>TAT	AAT – 13<br>TAT – 0	AAT – 810<br>TAT – 2	AAT – 1428<br>TAT – 2	AAT – 2034<br>TAT – 293	AAT – 1498<br>TAT – 1	AAT – 2855<br>TAT – 29	AAT – 1354<br>TAT – 0	AAT – 2510<br>TAT – 14	AAT – 1193<br>TAT – 2	AAT – 282<br>TAT – 13	AAT – 1026<br>TAT – 0	AAT – 19<br>TAT – 180	AAT – 6<br>TAT – 726
D614G	23403A>G	23402<br>GAT>GGT	GAT – 3<br>GGT – 158	GAT – 8<br>GGT 6668	GAT – 4<br>GGT 811	GAT – 0<br>GGT – 5299	GAT – 6<br>GGT – 904	GAT – 3<br>GGT – 2863	GAT – 14<br>GGT – 886	GAT – 3<br>GGT – 1560	GAT – 6<br>GGT – 702	GAT – 1<br>GGT – 26	GAT – 10<br>GGT – 827	GAT – 2<br>GGT – 173	GAT – 3<br>GGT – 834
R682W	23606C>T	23606<br>CGG>TGG	CGG – 98<br>TGG – 1	CGG – 1831<br>TGG – 4	CGG – 189<br>TGG – 407	CGG – 27<br>TGG – 0	CGG – 618<br>TGG – 5	CGG – 59<br>TGG – 0	CGG – 607<br>TGG – 3	CGG – 124<br>TGG – 0	CGG – 523<br>TGG – 6	CGG – 12<br>TGG – 1	CGG – 471<br>TGG – 5	CGG – 40<br>TGG – 4	CGG – 424<br>TGG – 3
D796Y	23948G>T	23948<br>GAT>TAT	GAT – 16<br>TAT – 70	GAT – 4293<br>TAT – 16	GAT – 1155<br>TAT – 0	GAT – 1235<br>TAT – 477	GAT – 1409<br>TAT – 5	GAT – 568<br>TAT – 649	GAT – 1313<br>TAT – 10	GAT – 97<br>TAT – 791	GAT – 38<br>TAT – 1257	GAT – 7<br>TAT – 0	GAT – 6<br>TAT – 1438	GAT – 2<br>TAT – 11	GAT – 12<br>TAT – 1223
A1078V	24795C>T	24974<br>GCT>GTT	GCT – 219<br>GTT – 3	GCT – 772<br>GTT - 4833	GCT – 772<br>GTT - 358	GCT – 4397<br>GTT – 1	GCT – 1319<br>GTT – 18	GCT – 2519<br>GTT – 12	GCT – 1270<br>GTT – 45	GCT – 1462<br>GTT – 30	GCT – 1418<br>GTT – 85	GCT –13695<br>GTT – 74	GCT – 1418<br>GTT – 36	GCT – 286<br>GTT – 17	GCT – 1264<br>GTT – 29

*Only deletions where the adjacent codon was preserved were counted.

3 Figure S1: Spike specific antibody levels with time post-SARS-CoV-2 diagnosis related to Figure 2.

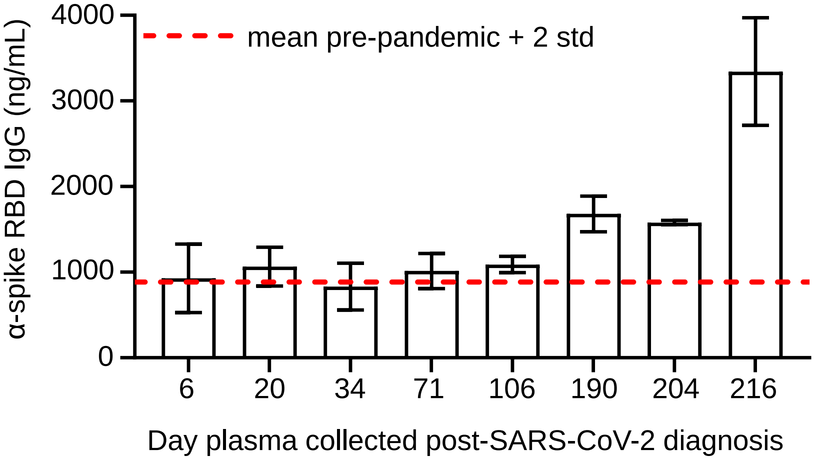

Fig S 1: Spike specific antibody levels with time post-SARS-CoV-2 diagnosis. Shown are mean (n=4 replicates per timepoint) and standard deviation of anti-spike RBD antibody concentrations measured in the plasma of the participant with advanced HIV disease by ELISA. Red dashed line denotes the mean + 2 standard deviations of signal from a set of 6 control samples, including plasma of pre-pandemic controls (n=4) and pre-pandemic commercial human serum (n=2).

4 Figure S2: Neutralization of Beta variant and evolved virus by a subset of BNT162b2 plasma donors related to Figure 2

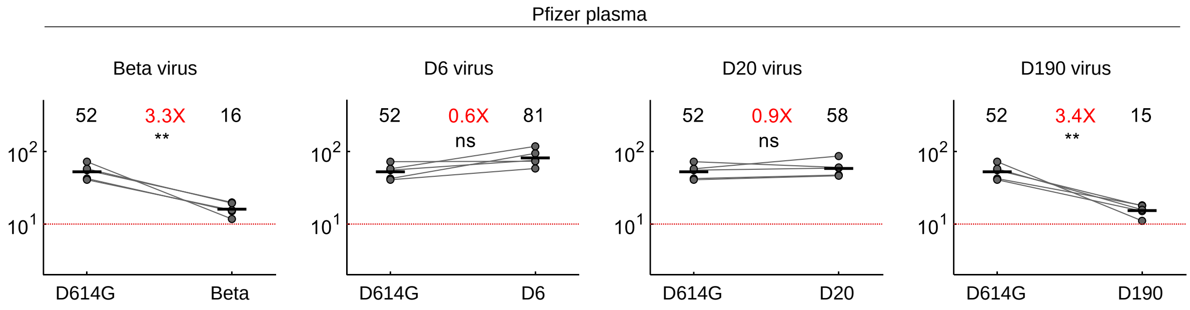

Fig S 2: Neutralization of Beta variant and evolved virus by a subset of BNT162b2 plasma donors. Neutralization of Beta, D6, D20, and D190 compared to D614G by Pfizer BNT162b2 plasma (n=5). Plasma donors were 136072, 136074, 136075, 136076, 136078. Red horizontal line denotes most concentrated plasma tested. Numbers in black are GMT FRNT50. Numbers in red are fold-change in GMT between virus strain on left and right. ** <0.01-0.001, as determined by the Wilcoxon rank sum test.
